# Sulfur Amino Acid Restriction alters mitochondrial function depending on tissue, sex, and Methionine sulfoxide reductase A (MsrA) status

**DOI:** 10.21203/rs.3.rs-3755231/v1

**Published:** 2023-12-21

**Authors:** Kevin M. Thyne, Raechel Camones, Adam B. Salmon

**Affiliations:** The University of Texas Health Science Center at San Antonio

**Keywords:** metabolism, reactive oxygen species, kidney, liver, longevity

## Abstract

Methionine restriction (MR) has been shown to affect mitochondrial function including altering oxygen consumption, reactive oxygen species (ROS) generation, Complex expression, and oxidative damage. The sulfur-containing amino acid methionine can become oxidized forming methionine sulfoxide which can lead to changes in protein function and signaling. Methionine sulfoxide reductases are endogenous enzymes capable of reducing methionine sulfoxide, with Methionine sulfoxide reductase A (MsrA) being ubiquitously expressed in mammals. Here we investigated if the effects of MR on mitochondrial function required functional MsrA in the liver and kidney which are the major tissues involved in sulfur biochemistry and both highly express MsrA. Moreover, MsrA is endogenously found in the mitochondria thereby providing potential mechanisms linking diet to mitochondrial phenotype. We found sex-specific changes in oxygen consumption of isolated mitochondria and females showed changes with MR in a tissue-dependent manner – increased in liver and decreased in kidney. Loss of MsrA increased or decreased oxygen consumption depending on the tissue and which portion of the electron transport chain was being tested. In general, males had few changes in either tissue regardless of MR or MsrA status. Hydrogen peroxide production was increased in the kidney with MR regardless of sex or MsrA status. However, in the liver, production was increased by MR in females and only slightly higher with loss of MsrA in both sexes. Mitochondrial Complex expression was found to be largely unchanged in either tissue suggesting these effects are driven by regulatory mechanisms and not by changes in expression. Together these results suggest that sex and MsrA status do impact the mitochondrial effects of MR in a tissue-specific manner.

## Introduction

There has been a long appreciation of the relationship between aging and a loss of mitochondrial function, including bioenergetic, biophysical, and replicative properties ^1^. In addition to being considered a “hallmark of aging” ^1^, deficits in mitochondrial homeostasis also directly affect other processes associated with aging including cellular senescence, inflammation, and energetics ^2–4^. Many longevity interventions have been reported to drive beneficial change to mitochondrial function ^5–7^. Methionine restriction (MR) has been shown to extend longevity and directly impact mitochondrial function ^8–18^. In rodent models, MR has been established to decrease mitochondrial reactive oxygen species (ROS) generation in different tissues in conjunction with decrease mitochondrial DNA oxidation ^8,14^, despite increased oxygen consumption ^8,13–18^. While the beneficial effects of MR, including improved metabolic function and longevity, may be driven partially by its effects on mitochondria, the mechanisms by which these are regulated have yet to be determined and could be developed as targets for novel interventions to improve healthy aging.

Our group has explored the potential role that methionine sulfoxide reductase A (MsrA), a regulator of methionine oxidation/reduction (redox), may play in the molecular and physiological effects of MR ^19,20^. The chemical structure of methionine is easily oxidized to methionine sulfoxide which has been shown to modify protein structure and function ^21–25^. MsrA and other methionine sulfoxide reductases have evolved to repair this oxidative damage ^26–29^ and provide cellular protection from oxidative stress ^30–32^. There is growing evidence that MsrA may regulate some functional properties of mitochondria ^33^, though because MsrA is localized in both cytosol and mitochondria it is not clear if these are direct or indirect actions of the enzyme ^34^. In yeast, lack of MsrA reduces mitochondrial efficiency and increases ROS production suggesting that MsrA is required for normal mitochondrial function ^35^. Similarly, mitochondria from the muscle of mice lacking MsrA (MsrA KO) have been shown to have bioenergetic deficits under normal conditions ^36^ while studies from an Alzheimer’s Disease mouse model lacking MsrA showed decreased Complex 4 activity and oxygen consumption in brain-derived samples ^37^. In cell based models, knockdown of MsrA was shown to reduce mitochondrial ATP content and reduce Complex 4 activity while increasing expression of MsrA had the reverse effect ^38^. Taken together these studies indicate a regulatory or functional role between MsrA and mitochondrial function.

Previously we showed that MsrA was not required to mediate the beneficial physiological effects of MR on glucose metabolism and body weight/composition. In these studies, we found that the lack of MsrA in mice manifested greater loss in lean and fat mass with MR, as well as larger improvements to glucose metabolism relative to normal diet compared to that in control mice ^19^. These results suggest MsrA has an uncharacterized role in mediating or modulating MR. In this study we investigated if MsrA was directly involved in the regulation of mitochondrial function in response to MR. We specifically tested the response of mitochondria in bioenergetics, physiology, and complex composition in multiple tissues from mice under MR and in the presence/absence of MsrA. Overall, our reports point to tissue- and sex-dependent effects of MR on mitochondrial respiration and generation of oxygen radicals, and hints at interactions with MsrA directly. Overall, our studies add to the growing evidence for MsrA in maintaining normal mitochondrial homeostasis and a potential role for this enzyme in mediating the molecular effects of MR in the liver and kidney.

## Methods

### Animal Usage and Ethical Procedures

All animal experiments were approved by the Institutional Animal Care and Use Committees and UTHSA (Animal Protocol 20170190AR), and have been reported following ARRIVE guidelines. All methods were conducted in accordance with international ethical standards and guidelines.

### Animal use and diets

We have described all husbandry conditions, the genetic mutant MsrA KO mouse, and diets used in this study in our previous publications ^19,20,39^. All mice were maintained in the C57Bl/6J background and MR diets used were control diet (CD) (0.86% Met, TestDiet 578F w/0.86% MET – 5SFD) or MR (0.15% Met, 0% Cys, Test Diet 96D2, modified TestDiet 58B0). 7–10 mice were assigned randomly to each group and the age range at time of enrollment was 11.4 to 14.3 months old with average age similar for all groups. Mice were fed each diet for approximately 2 months before the end of the study and were euthanized via CO_2_ asphyxiation at which time tissues were collected for isolated mitochondrial studies. Tissue samples collected from an independent group of animals reported previously ^19^ were used for frozen mitochondrial assays and immunoblots.

### Mitochondrial Isolation

Fresh liver and kidney were collected and rinsed twice with ice cold Mitochondrial Isolation Buffer (MSHE) (70 mM Sucrose, 210 mM Mannitol, 5 mM HEPES, 1 mM EGTA, pH 7.2 with KOH) to remove as much blood as possible from the samples. The buffer was decanted and the tissue samples were minced in a petri dish on ice with a razor blade until the tissue pieces reached approximately a few millimeters per dimension. The minced tissues were then disrupted by hand with a Dounce homogenizer on ice in 10 ml of MSHE. The disrupted tissue was transferred to a centrifuge tube and spun at 800 g for 10 min at 4°C. The top layer containing fat was aspirated with a pipette, the supernatant was removed with a pipette and strained through synthetic cheese cloth into a new tube. Samples were spun at 8000 g for 10 min at 4°C. The supernatant was decanted and the pellet was carefully resuspended in 25 ml of ice cold MSHE. This sample was centrifuged at 8000 g for 10 min at 4°C. The supernatant was decanted and the pellet of isolated mitochondria was resuspended in 60–200 μl MSHE. Concentration was measured by the Bradford method using a spectrophotometer. Isolated mitochondria were kept on ice and immediately used for oxygen consumption assessment and H_2_O_2_ generation assay.

### Oxygen Consumption Rate of Isolated Mitochondria

The Oxytherm (Hansatech Instruments) reaction chamber containing a Clark electrode was pre-filled with 0.5 ml of assay buffer (MHPM) (120 mM KCl, 10 mM KH_2_PO_4_, 2 mM MgCl_2_, 5 mM HEPES, 1 mM EGTA, pH 7.2 at 37°C) containing 0.3% defatted BSA warmed to 37°C minus the volume of the isolated mitochondrial sample. The isolated mitochondria were diluted 1:1 with the MHPM buffer containing 0.3% defatted BSA to a final concentration of 5–10 μg/μl. The Oxytherm chamber was allowed to equilibrate with the MHPM buffer and 0.5 μM Rotenone with the chamber closed. Then 75 μg of the diluted isolated mitochondria were added resulting in a final chamber volume of 500 μl which was then allowed to equilibrate for 2–5 min. After equilibration, succinate was added to 5 mM (5 μl of 500 mM stock) to drive State 2 respiration and once curve linearity was established (2–3 min) ADP was added to 0.3 mM (10 μl of 15 mM stock) to drive State 3 respiration (2–3 min). Results were graphed and maximum slope was determined for State 2 and State 3 by best-fit over a 20 second time period. RCR was calculated as the ratio of State 3 to State 2. Samples in which no response was shown to ADP were interpreted as being hyper-permeabilized and were censored from the dataset.

### Hydrogen Peroxide (H_2_O_2_) Production of isolated mitochondria

Reaction buffer master mix was prepared in MHPM with 0.3% defatted BSA containing saturating amounts of superoxide dismutase (SOD; 35–50 U/ml), 5 U/ml horseradish peroxidase (HRP), and 50 μM Amplex UltraRed (A36006, Invitrogen). Isolated mitochondria were added to a final concentration of 0.15 μg/ml and distributed in technical replicate 200 μl reactions in a clear, flat-bottom, black-walled 96-well plate (30 μg of isolated mitochondria per well). 2 μl of fresh 10 mM PMSF in 200 proof ethanol was added to each well (0.1 mM final concentration) and fluorescence was measured at 540 nm excitation/590 nm emission every minute in a 37°C plate reader (Molecular Devices SpectraMax M2) for 10 min to establish baseline. The plate was removed and 2 μl of 500 mM succinate was added (5 mM final concentration). Wells were mixed by pipetting and the plate was returned to the plate reader for an additional 10–11 min of reads at 1 min intervals. The plate was again removed and 2 μl of 400 uM rotenone was added (4 μM final concentration). Wells were mixed by pipetting and the plate was returned to the plate reader for an additional 10–11 min of reads at 1 min intervals. Results were graphed and instantaneous slopes measured for the first 10 data points (10 min) of each segment of each run and corrected for baseline H_2_O_2_ production rate. Instantaneous rate was the average of the slopes for pairs of neighboring points which was more resistant to skewing by aberrant data points with tended to result in significant changes to best-fit calculations given the small number of data points.

### Mitochondrial Oxygen Consumption from Frozen Tissue

Sample prep and assays were performed using the methods of by Osto, et al.^40^ Briefly, ~ 50 mg of frozen tissues were homogenized at 10:1 ratio (buffer to tissue) in 1x MAS buffer (70 mM Sucrose, 220 mM Mannitol, 5 mM KH_2_PO_4_, 5 mM MgCl_2_, 1 mM EGTA, 2 mM HEPES, pH 7.4 using KOH) using a TissueLyser Lite (Quiagen) at 35 Hz for 70 sec. The homogenate was then centrifuged at 200 g for 5 min at 4 C and supernatant was collected. Total protein concentration was measured via BCA assay (Pierce). Samples were diluted in 1x MAS buffer to 1.0 μg/μl for use in assays. XF96 Seahorse Extracellular Flux plates (Seahorse XFe96/XF Pro FluxPac, 103792–100, Agilent) were prepared and calibrated as per manufacturer’s instructions. The plate was equilibrated and measured at 28°C. 20 μl of the 1.0 μg/μl liver homogenate were added to the tissue plate (20 μg total protein) and allowed to incubate on ice for 52 min while the injection ports were calibrated. The tissue plate loaded with the mitochondria was then centrifuged at 2000 × g for 5 min at 4 C without centrifuge breaking enabled (25 min total cycle time). 130 μl of ice cold 1x MAS buffer with 10 μg/ml Cytochrome C was added to each well. The tissue plate was then tested with the protocol indicated by Osto, et al.^40^

### Western Blot

Approximately 50 mg of frozen tissue were homogenized in ~ 500 μl RIPA (10 μl RIPA: 1 mg tissue) with protease and phosphatase inhibitors (Pierce). Tissue samples were homogenized using a TissueLyserII (Quiagen) for 1 min at 30 Hz for liver. Supernatant after centrifugation was collected and protein concentration measured using Pierce BCA assay kit (Bio-Rad). Blotting was performed using Criterion TGX gels (Bio-Rad) followed by transfer to PVDF membranes (Bio-Rad). Total protein was measured with Ponceau S (Sigma) staining imaged with a Perfection V39 flatbed scanner (Epson). Membranes were blocked with 10% non-fat dry milk in TBST. Membranes were incubated with primary antibody overnight at 4°C with agitation. The primary antibody was prepared in TBST with 2%BSA and 0.01% sodium azide. Antibody used: Total OxPhos/MitoProfile (MitoSciences, MS604/AbCam, ab110413). Membranes were washed with TBST and incubated with HRP secondary (Santa Cruz Biotechnology) for 1hr at room temperature. Membranes were then washed with TBST and developed with Pierce ECL Plus Western Blotting Substrate (ThermoFisher). Membranes were imaged on a Typhoon FLA 7000 (Amersham). All quantifications were performed in ImageStudioLite (Li-Cor). Quantifications were normalized to total protein. A standard sample was used for normalization and comparison between membranes.

### Statistics

Area Under the Curve was calculated by the trapezoid method in Excel. Best-fit (Oxytherm) and Instantaneous Rate (H_2_O_2_ Production) were calculated in Excel. Analyses were performed in Prism8. Results were analyzed by 2-Way ANOVA with Sidac multiple comparison corrections within each sex. Post-hoc testing was done to assess diet effect. 3-Way ANOVAs were completed to assess sex-effects without post-hoc testing.

## Results

**Figure 1:**
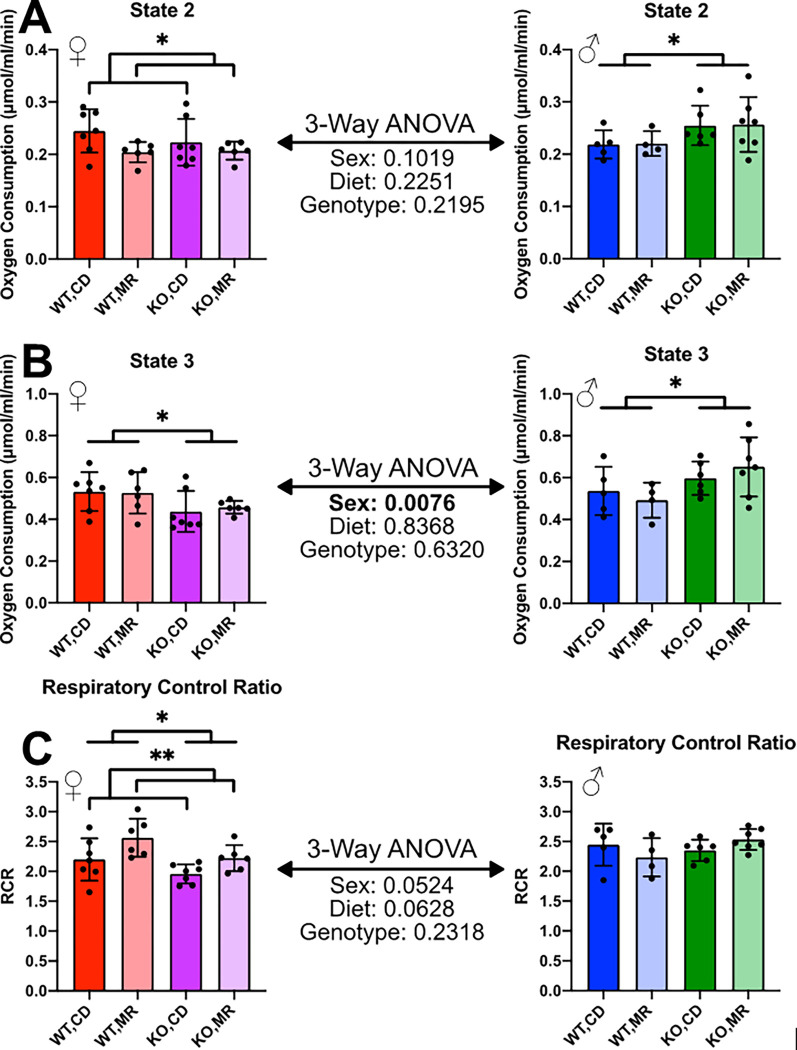
Kidney Isolated Mitochondria Oxygen Consumption Rate Oxygen Consumption Rate (OCR) was measured via Oxytherm Clark electrode. Isolated mitochondria were allowed to stabilize oxygen consumption in the presence of rotenone. Succinate was used as substrate for State 2 respiration (**A**), followed by the addition of ADP for State 3 respiration (**B**). Respiratory Control Ratio (RCR) was calculated as the ratio of State 3 to State 2. Analysis was completed within each sex via 2-Way ANOVA for main effects with post-hoc analysis performed with Sidac multiple comparisons correction to assess the diet effect within each genotype. 3-Way ANOVA was performed to assess sex-effect. Graphs represent mean ± SD. Group sizes were 4–7. (* p < 0.05; ** p < 0.01; *** p <0.001)

For isolated mitochondria studies, liver and kidney were processed immediately following collection from MR-treated mice and controls. Oxygen consumption rate (OCR) measured in isolated kidney mitochondria indicated that State 2 respiration (respiration in the presence of substrate but not ADP) was significantly reduced by MR in females, but there was no significant effect in males. The lack of MsrA was associated with higher respiration compared to controls in males regardless of diet while this effect was not present in females (Fig. 1A). In contrast, State 3 respiration (respiration in the presence of substrate and ADP) was unaffected by MR, but was significantly affected by lack of MsrA in disparate ways in males and females. State 3 OCR was decreased in females but increased in males (Fig. 1B). Respiratory control ratio (RCR), the ratio of State 3 to State 2 respiration, was increased by MR in females only. Additionally, lack of MsrA was associated with decreased RCR in females. RCR in males was unaffected by MR or MsrA status (Fig. 1C).

**Figure 2:**
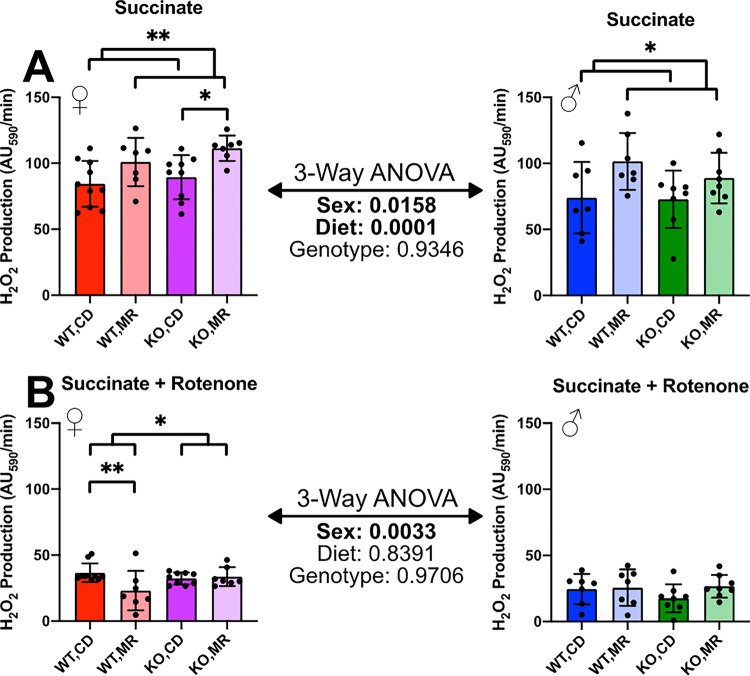
Kidney Isolated Mitochondria H_2_O_2_ Production H_2_O_2_ production of kidney isolated mitochondria measured by AmplexRed Ultra fluorescence assay with succinate as substrate (**A**) followed by the addition of rotenone (**B**). Analysis was completed within each sex via 2-Way ANOVA for main effects with post-hoc analysis performed with Sidac multiple comparisons correction to assess the diet effect within each genotype. 3-Way ANOVA was performed to assess sex-effect. Graphs represent mean ± SD. Group sizes were 7–10. (* p < 0.05; ** p < 0.01)

We assessed these kidney mitochondria for H_2_O_2_ production since previous studies have shown significant reduction of mitochondrial reactive oxygen species (ROS) production with MR in various tissues ^8,14–18^. Surprisingly, in our kidney-derived samples, MR was associated with increased H_2_O_2_ production in the presence of succinate as Complex 2 substrate in both sexes with 3-Way ANOVA indicating a sex-effect in which females had greater production than males (Fig. 2A). The addition of rotenone to prevent H_2_O_2_ generation via electron back-feeding from Complex 2 to Complex 1 showed a large decrease in H_2_O_2_ production rate in both sexes and loss of the MR main effect (Fig. 2B). Despite this drop in H_2_O_2_ production, females still had higher H_2_O_2_ production as indicated by the 3-Way ANOVA results. In females the wild type mice showed a decrease in H_2_O_2_ production in response to MR while the MsrA KO did not receive any effect.

**Figure 3:**
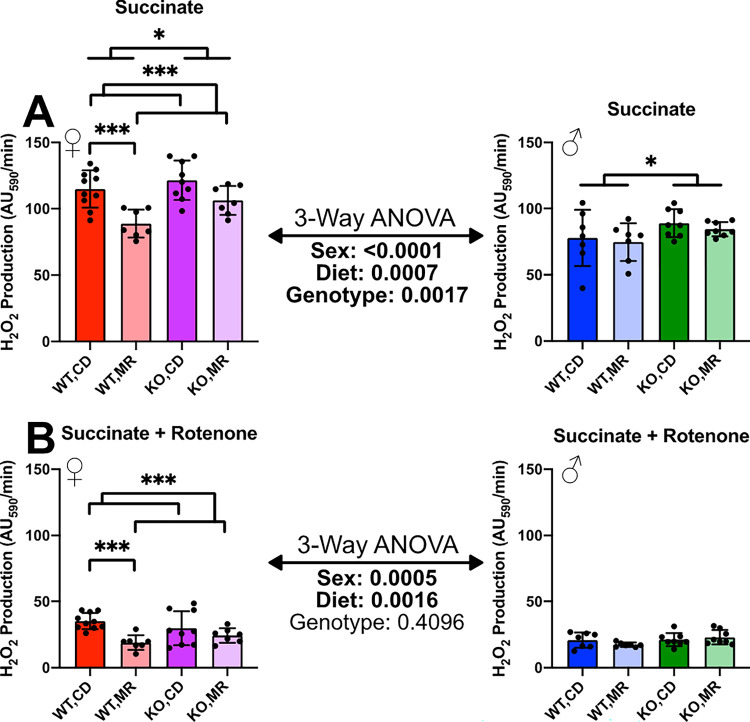
Liver Isolated Mitochondria H_2_O_2_ Production H_2_O_2_ production of kidney isolated mitochondria measured by AmplexRed Ultra fluorescence assay with succinate as substrate (**A**) followed by the addition of rotenone (**B**). Analysis was completed within each sex via 2-Way ANOVA for main effects with post-hoc analysis performed with Sidac multiple comparisons correction to assess the diet effect within each genotype. 3-Way ANOVA was performed to assess sex-effect. Graphs represent mean ± SD. Group sizes were 7–10. (* p < 0.05; ** p < 0.01; *** p < 0.001)

Contrary to those from the kidney, mitochondria isolated from the liver show a significant decrease in succinate-stimulated H_2_O_2_ production in MR-treated females, though MR had no effect in males. For both sexes, the lack of MsrA is associated with increased H_2_O_2_ production relative to wild type mice. 3-Way ANOVA indicated a sex-effect with females having higher H_2_O_2_ production than males (Fig. 3A). The addition of rotenone decreased H_2_O_2_ production, however females still had higher H_2_O_2_ production as indicated by the 3-Way ANOVA. Under these conditions, MR was associated with decreased H_2_O_2_ production in the females while there was no effect in the males. Additionally, MsrA status has no effect in females or males (Fig. 3B).

**Figure 4:**
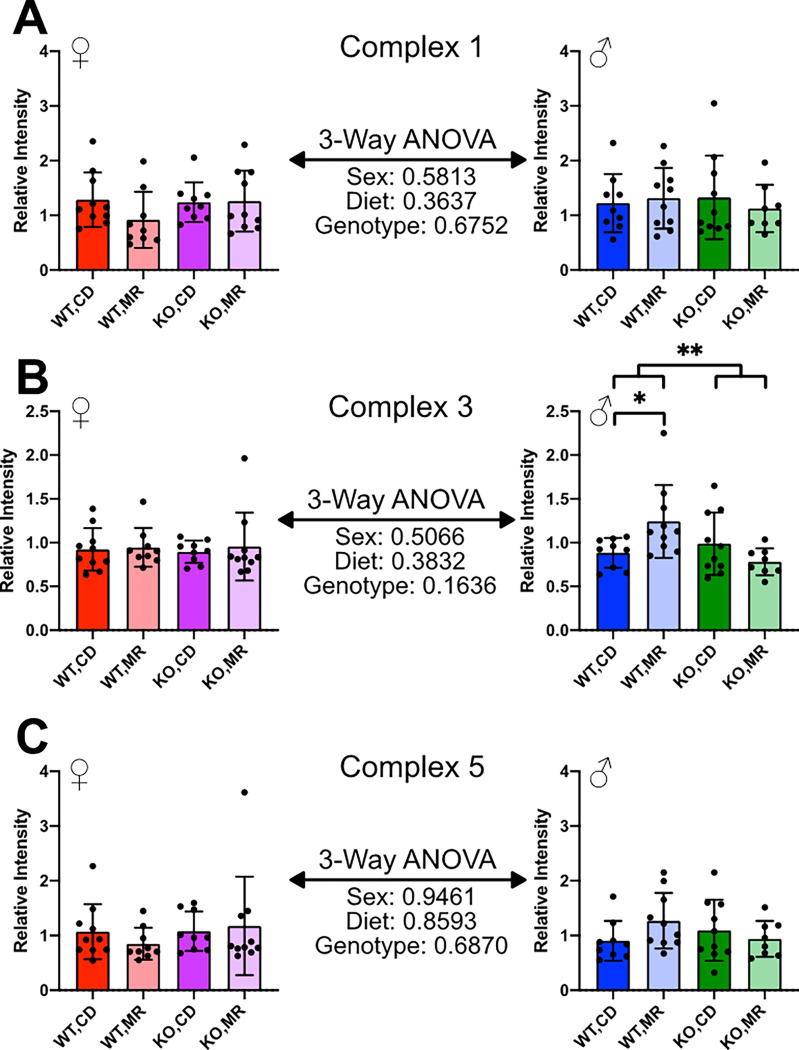
Kidney Mitochondrial Complex Expression Samples from cohort 2 were used to measure the expression of Mitochondrial Complexes in liver tissue homogenates via Western blot. Complex 1 (**A**), Complex 3 (**B**), and Complex 5 (**C**) were measured. Sexes were analyzed separately via 2-Way ANOVA. A 3-Way ANOVA was performed to compare between sexes for main effects. Graphs represent mean ± SD, group sizes were 7–10 mice.

**Figure 5:**
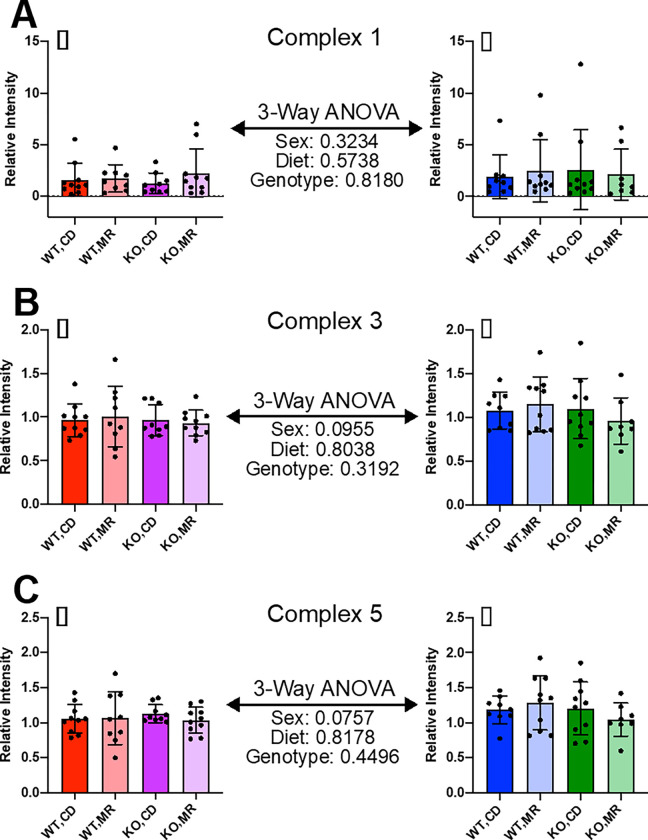
Liver Mitochondrial Complex Expression Samples from cohort 2 were used to measure the expression of Mitochondrial Complexes in kidney tissue homogenates via Western blot. Complex 1 (**A**), Complex 3 (**B**), and Complex 5 (**C**) were measured. Sexes were analyzed separately via 2-Way ANOVA. A 3-Way ANOVA was performed to compare between sexes for main effects. Graphs represent mean ± SD, group sizes were 7–10 mice.

To begin to address potential molecular mechanisms for these differences in mitochondrial physiology among sex, diet, and genotype, we first addressed whether there was evidence for global changes in the expression of the electron transport chain complexes. In tissue lysates from liver and kidney, we found no significant effect of sex, genotype, or MR on expression of Complex 1, 3, and 5, with the exception of increased Complex 3 expression in the kidney of male wild-type mice on MR (Fig. 4, Fig. 5).

**Figure 6:**
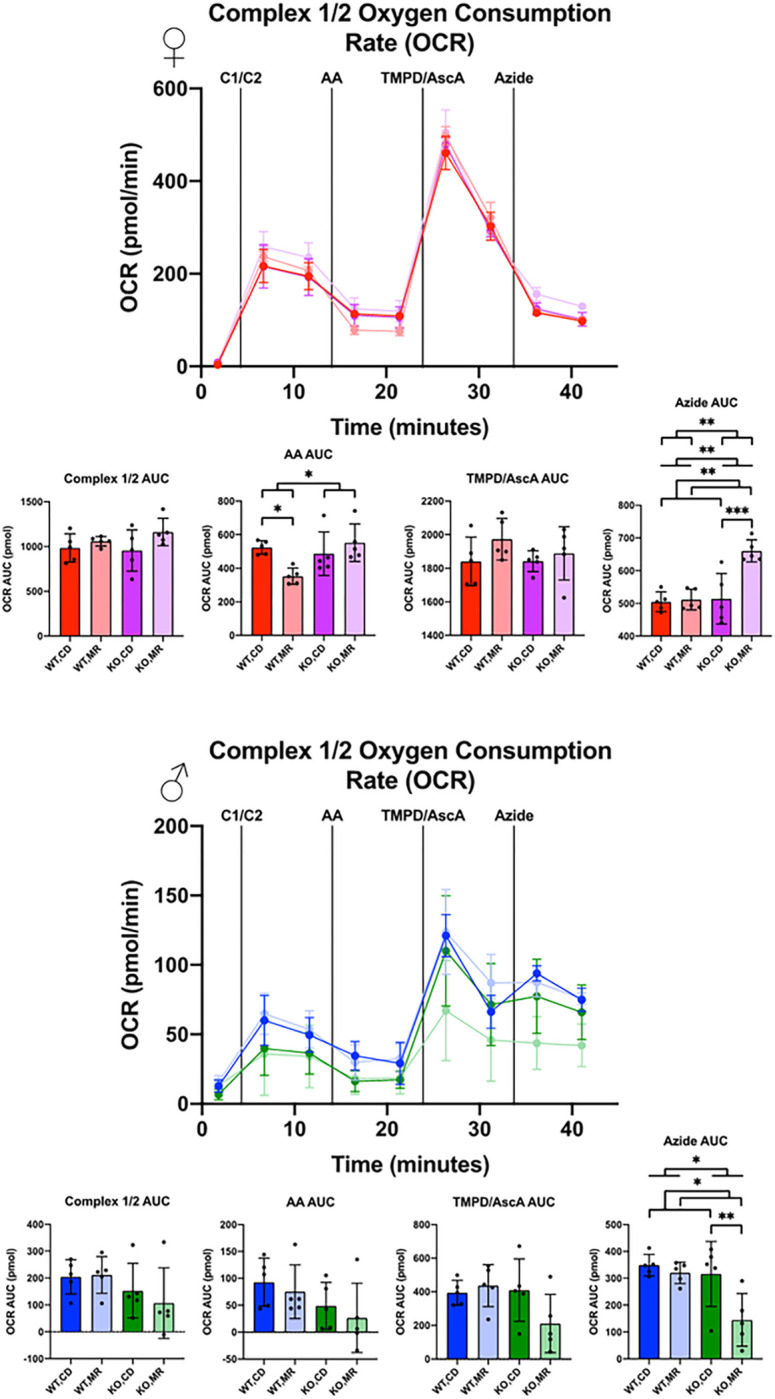
Liver Isolated Mitochondria Oxygen Consumption Rate, Complex 1/Complex 2 Liver isolated mitochondria from frozen tissue were measured for Oxygen Consumption Rate (OCR) via 96-well Seahorse. 10μg of isolated mitochondria were used in MAS buffer in the presence of 10μg/ml Cytochrome C. Injections were as follows: Complex 1/Complex 2 OCR using NADH as substrate (C1/C2), Complex 3 inhibition with Antimycin A (AA), Complex 4 OCR with N,N,N’,N’-tetramethyl-*p*-phenylenediamine and Ascorbic Acid (TMPD/AscA), Complex 4 Inhibition with Sodium Azide (Azide). Area Under the Curve (AUC) was calculated via the trapezoid method for each segment. Analysis was completed within each sex via 2-Way ANOVA for main effects with post-hoc analysis performed with Sidac multiple comparisons correction to assess the diet effect within each genotype. Graphs represent mean ± SD. Group sizes were 5. (* p < 0.05; ** p < 0.01)

**Figure 7:**
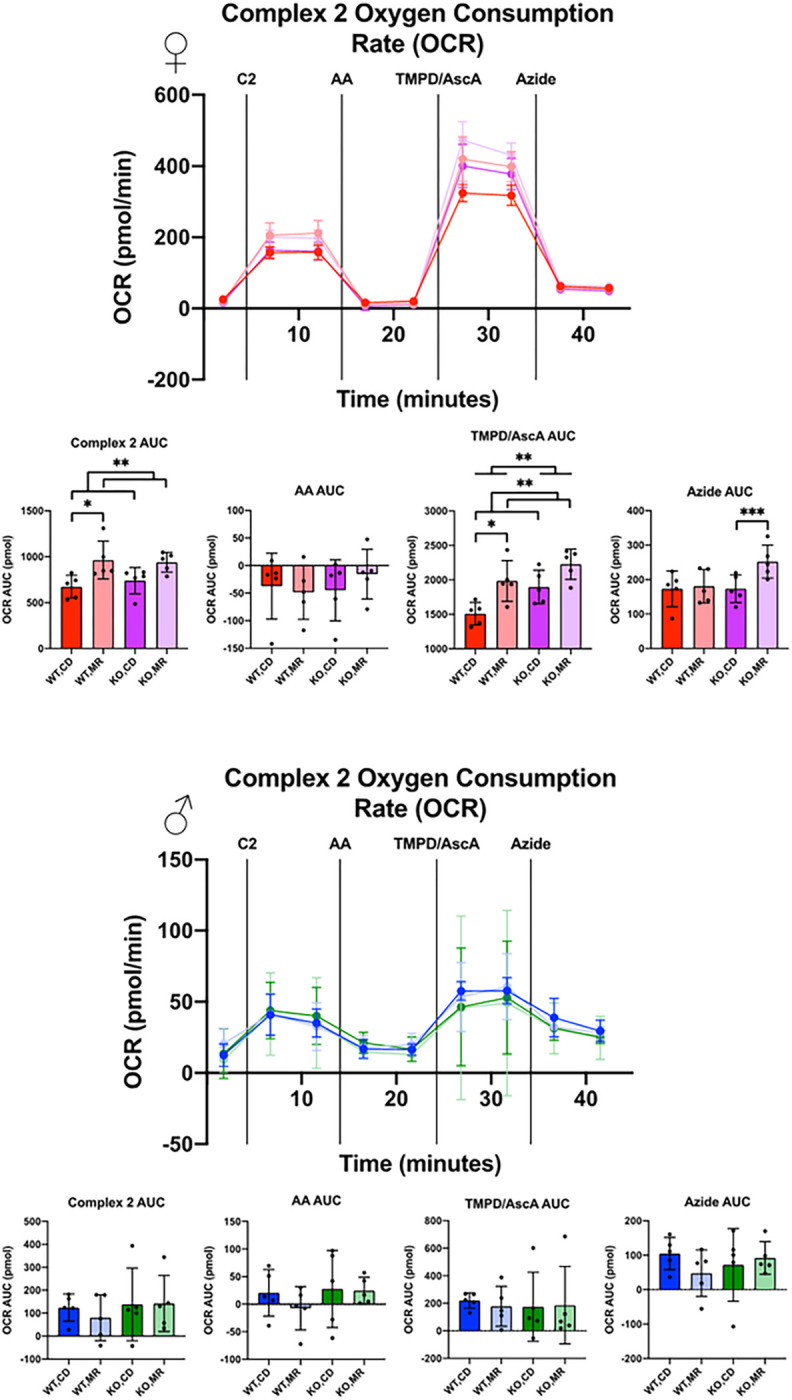
Liver Isolated Mitochondria Oxygen Consumption Rate, Complex 2 Liver isolated mitochondria from frozen tissue were measured for Oxygen Consumption Rate (OCR) via 96-well Seahorse. 10μg of isolated mitochondria were used in MAS buffer in the presence of 10μg/ml Cytochrome C. Injections were as follows: Complex 2 OCR using Succinate and Rotenone as substrate and Complex 1 inhibitor (C2), Complex 3 inhibition with Antimycin A (AA), Complex 4 OCR with N,N,N’,N’-tetramethyl-*p*-phenylenediamine and Ascorbic Acid (TMPD/AscA), Complex 4 Inhibition with Sodium Azide (Azide). Area Under the Curve (AUC) was calculated via the trapezoid method for each segment. Analysis was completed within each sex via 2-Way ANOVA for main effects with post-hoc analysis performed with Sidac multiple comparisons correction to assess the diet effect within each genotype. Graphs represent mean ± SD. Group sizes were 5. (* p < 0.05; ** p < 0.01)

Using a method to measure mitochondrial respiration from whole tissue homogenates generated from frozen samples ^40^, we addressed specific mitochondrial complex activity using samples generated from frozen liver homogenates (Fig. 6, Fig. 7). In samples from males, there was largely no effect of MsrA or MR on Complex 1/2 or Complex 2 activity aligning largely with our results from isolated mitochondria. OCR of Complex 1/2 in females showed minimal effects of MR or MsrA with the exception of block of Complex 3 with Antimycin A (AA) which resulted in an interaction effect (Fig. 6). In females, we found that MR increased OCR of Complex 2 substrates with MsrA having no effect no outcome. Blocking of Complex 3 with Antimycin A (AA) largely eliminated these differences, suggesting that the differences in OCR with substrate was driven by the level of coupling between the complexes. Testing of Complex 4 with TMPD/Ascorbic Acid (TMPD/AscA) showed a substrate dependent effect in the females in which MR and MsrA KO both caused increased OCR, suggesting either greater Complex 4 expression or more efficient function. Complex 5 inhibition with azide showed substrate dependent effects, but was highly variable. Within-sex groups were generally similar with the exception of the MsrA KO group on MR (Fig. 7). The exact mechanism for this is unclear. It is also unclear why the female OCR was significantly higher than the males in the liver. ECAR was also determined for all runs and with the exception of some diet effects in the wild-type control males no other effects were observed (Supplemental Fig. 1, Supplemental Fig. 2).

## Discussion

Previous studies have shown MR to have a variety of impacts on mitochondrial function ^8,13–18,41,42^. Here we expand on these findings and point to sex- and tissue-specific differences in mitochondrial response to MR as well as potential roles for methionine oxidation repair via MsrA in this process. Moreover, we demonstrate that different tissues within the same donor respond differently to MR. In particular, we show that MR increased H_2_O_2_ production from mitochondria isolated from the kidney and decreased this output in mitochondria isolated from the liver (Fig. 2, Fig. 3). The mechanisms and consequences for this disparity are still unknown, but important to consider when considering the potential for interventions like MR in benefiting health.

In this study, we found that males tended to have blunted response to MR than females in terms of mitochondrial function. This is interesting in light of our previous studies on physiological changes in response to MR which showed that males tended to benefit more in terms of physiological measures and glucose homeostasis ^19^. In this study the mice also responded similar to treatment in terms of body weight and food consumption (Supplemental Fig. 3). These findings raise interesting questions regarding the mechanistic link between improved metabolism at the organismal level with MR and mitochondrial function. However, further exploration of other tissues important for glucose regulation, including muscle, adipose, and pancreatic β-cells would likely be required to better address this question. It is also important to note that many of these experiments were performed with succinate as a substrate and that different substrate/inhibitor combinations may change these outcomes.

Overall our data show that MR tends to have mixed effects on mitochondrial H_2_O_2_ production that are dependent on both sex and, to some degree, the presence of MsrA. MR had a pronounced effect on H_2_O_2_ production in the kidney with it being increased in the presence of succinate, but this increase was lost in the presence of rotenone (Fig. 2). This is unexpected since MR generally decreases or has no effect on H_2_O_2_ production although this has been demonstrated in tissues other than the kidney ^8,14–16,18,42^. The liver results were more in line with other published results with MR decreasing H_2_O_2_ production however this was only significantly changed in the females (Fig. 3). While MsrA largely had little effect on mitochondrial function overall, we did show some discreet effects of this enzyme on both mitochondrial function and the effect of MR. Interestingly, in kidney the absence of MsrA blocked the effect of MR on H_2_O_2_ production. With MsrA localized in the mitochondria endogenously ^34^, the enzyme could play an important role in the maintaining the mitochondrial proteome homeostasis ^43^. While more work is required, this raises an intriguing possibility that at least some mitochondrial physiology regulated by MR requires either reduction of methionine oxidative damage or regulation of methionine redox properties, both of which are driven by MsrA.

Comparisons between sexes showed some interesting dichotomies that warrant further exploration. In both kidney and liver, mainly females showed changes in respiration in response to MR as shown by increased OCR in the presence of complex 2 substrate for the liver, but decreased OCR for the kidney (Fig. 6, Fig. 7). The genotype effect present in the kidney OCR results was not as prevalent in the liver. It is also important to note that the female liver had significantly higher OCR than the males, regardless of the substrate tested – Complex 1/2 versus Complex 2. The reason for this large difference in OCR between the sexes is unclear. Data in the literature has shown that OCR can be different between sexes in various tissues, including the liver, however not to this degree ^44,45^. Mitochondrial complex expression was shown to be largely unchanged in either liver or kidney (Fig. 4, Fig. 5). These results are in line with existing literature indicating generally no change, although there are some exceptions ^13–18,41,46^. It may be that there are differences in supercomplex/respirasome formation that could account for this difference but would require additional investigation.

## Data Availability

The data presented in the work are available from the corresponding author upon request.
